# Clinical outcomes of arthroscopic single and double row repair in full thickness rotator cuff tears

**DOI:** 10.4103/0019-5413.65160

**Published:** 2010

**Authors:** Jong-Hun Ji, Mohamed Shafi, Weon-Yoo Kim, Young-Yul Kim

**Affiliations:** Department of Orthopedic Surgery, Daejon St. Mary’s Hospital, The Catholic University of Korea

**Keywords:** Shoulder arthroscopy, rotator cuff tear, single row repair, double row repair

## Abstract

**Background:**

There has been a recent interest in the double row repair method for arthroscopic rotator cuff repair following favourable biomechanical results reported by some studies. The purpose of this study was to compare the clinical results of arthroscopic single row and double row repair methods in the full-thickness rotator cuff tears.

**Materials and Methods:**

22 patients of arthroscopic single row repair (group I) and 25 patients who underwent double row repair (group II) from March 2003 to March 2005 were retrospectively evaluated and compared for the clinical outcomes. The mean age was 58 years and 56 years respectively for group I and II. The average follow-up in the two groups was 24 months. The evaluation was done by using the University of California Los Angeles (UCLA) rating scale and the shoulder index of the American Shoulder and Elbow Surgeons (ASES).

**Results:**

In Group I, the mean ASES score increased from 30.48 to 87.40 and the mean ASES score increased from 32.00 to 91.45 in the Group II. The mean UCLA score increased from the preoperative 12.23 to 30.82 in Group I and from 12.20 to 32.40 in Group II. Each method has shown no statistical clinical differences between two methods, but based on the sub scores of UCLA score, the double row repair method yields better results for the strength, and it gives more satisfaction to the patients than the single row repair method.

**Conclusions:**

Comparing the two methods, double row repair group showed better clinical results in recovering strength and gave more satisfaction to the patients but no statistical clinical difference was found between 2 methods.

## INTRODUCTION

The outcome of arthroscopic rotator cuff repair is now comparable to that of open repair with improvement in arthroscopic techniques. The rate of re-tear after arthroscopic repair ranges from 76 to 94%^1^ and for open or mini-open repair between 20 and 70%.^2^ Some studies have reported that it is crucial to establish a rotator cuff footprint to improve healing and the initial rotator cuff strength.^3^ After the introduction of the 3-dimensional reconstruction concept of the rotator cuff footprint by Apreleva *et al*^4^ a double row repair method that reconstructs the footprint, has been introduced. Many shoulder surgeons are currently using the double row repair technique because of the better biomechanical results reported by this technique.^5^,^6^,^7^ However, there are a few clinical reports on the outcome of the double row repair technique.^8^,^9^ The purpose of this study was to retrospectively compare the clinical outcome of the single row repair (Group I) and the double row repair (Group II) after arthroscopic cuff repair using suture anchors.

## MATERIALS AND METHODS

Between March 2003 to March 2005, 250 patients with a degenerative rotator cuff tear were treated by shoulder arthroscopy, out of which 47 patients with a complete rotator cuff tear, confirmed by MRI preoperatively, were enrolled in this study. Only patients with complete rotator cuff tears were included. Patients with partial, massive and irreparable cuff tears, as well as subscapularis and biceps tendon tears were excluded. The rotator cuff tendon could be mobilized to the greater tuberosity in all the patients which included in this study and the outcomes of the two repair techniques were compared. In the early part of the study, the patients mainly underwent arthroscopic rotator cuff repair using a single-row technique whereas in the latter part of the study, the double row technique was used. The patients were divided into two groups, 22 cases underwent the single row repair and were assigned to group I; 25 cases underwent the double row repair and were assigned to group II. The mean age of group I was 58 years (49-73 years) and that for group II was 56 years (43-72 years). There were 12 males and 10 females in group I and 17 males and 8 females in group II. The follow-up period for the postoperative functional evaluation in group I mean 22 (18-24 months) and in group II mean 24 (22-28 months). In both groups, we measured the mean anteroposterior (AP) size and the mediolateral (ML) size of the torn cuff. However, the anteroposterior tear size was more useful than the mediolateral tear size when choosing the number of inserted anchors for the footprint of the greater tuberosity. The preoperative tear size was assessed during arthroscopic surgery. Eight tears were small (less than 1 cm in length), 22 were medium (1 to 3 cm) and 17 were large (3 to 5 cm). There were 3 small, 10 medium and 9 large tears in the single-row group, and five small, 12 medium and eight large in the double row group. All the patients were evaluated by senior author (JHJ) using the University of California Los Angeles (UCLA)^10^ rating scale and the shoulder index of the American Shoulder and Elbow Surgeons (ASES)^11^ preoperatively and at the final follow-up examination.

### Operative procedure

**Figure 1:**
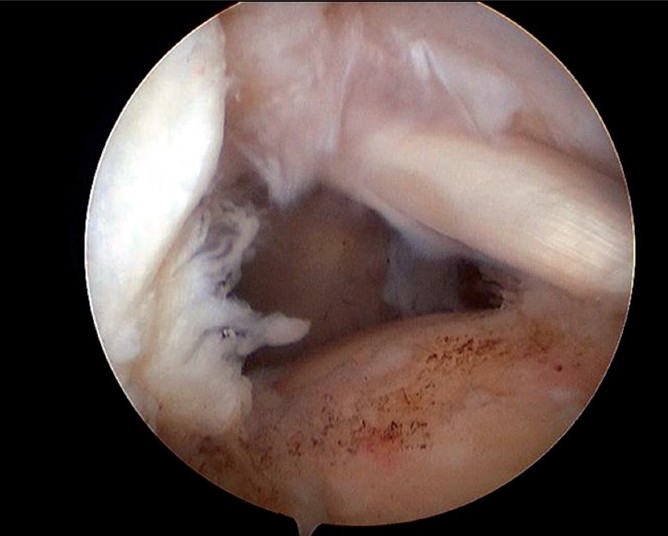
Arthroscopic view of left shoulder shows bare rotator cuff footprint area

The patients were operated under general anesthesia in the lateral position with standard posterior, lateral and postero-lateral arthroscopic portals. The diagnostic arthroscopy evaluated the glenohumeral joint including the torn cuff, articular capsule, subscapularis tendon and biceps tendon. For a chronic retracted tear, release of the postero-superior capsule from the glenoid side was performed with a shaver or electrocautery and thus the cuff tendon had increased mobility. After that, the arthroscope was moved to the subacromial space and any pathologic bursal tissue that impeded clearance of the space was removed; arthroscopic subacromial decompression was performed to create a flat acromial undersurface in all patients. Osteophytes in the inferior part of the acromioclavicular joint and the distal end of the clavicle were also removed if necessary. The footprint of the entire torn rotator cuff was visualized, a grasper pulled the torn cuff margin so that the mobility of the anterior-to-posterior side and the medial-to-lateral side could be evaluated [Figure 1]. For the double row technique, the most important part of the procedure is the possibility for pulling the torn cuff to the greater tuberosity of the humeral head. In addition, a coracohumeral ligament release was performed to improve cuff mobility in all patients.

**Figure 2:**
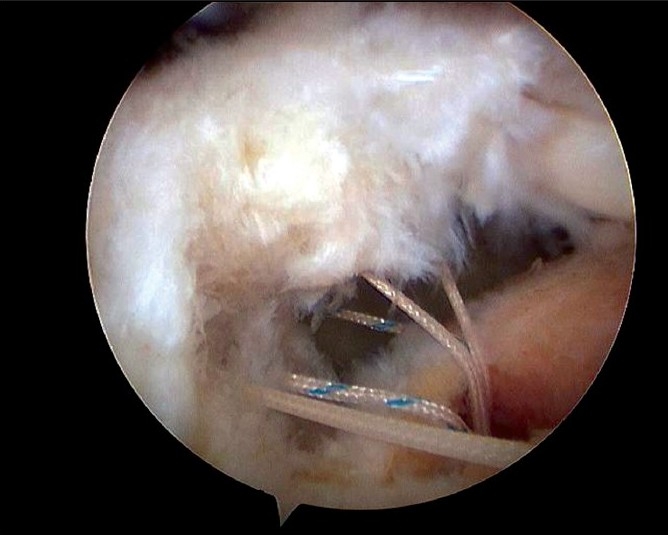
Arthroscopic image of left shoulder shows the medial-row anchors have been placed, and the sutures have been passed but not secured

**Figure 3:**
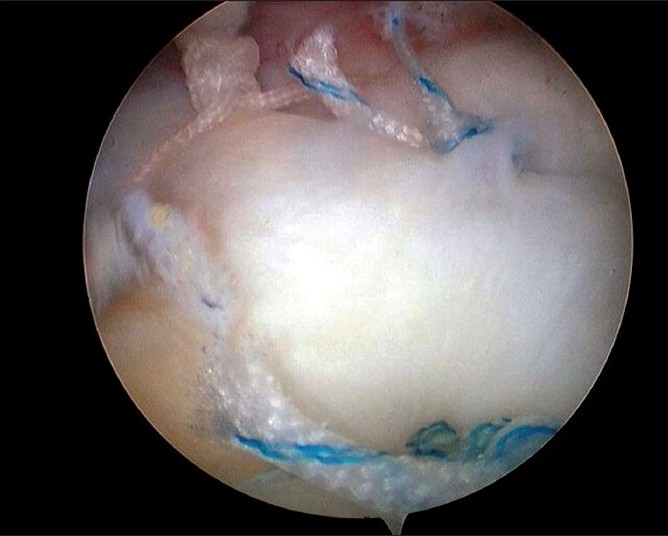
Arthroscopic image of same shoulder shows completed suture of medial row

Initially, medial row anchors (5.0mm TwinFix anchors, Smith and Nephew Endoscopy, Andover, MA) were inserted just lateral to the margin of the humeral head articular surface [Figure 2]. Depending on the anterior-posterior dimension of the torn cuff, one or two anchors were inserted into the medial row. Usually, the medial to lateral dimension of the footprint of the rotator cuff is about 16 mm,^12^ and thus by using the Scorpion suture passer (Arthrex, Naples, FL, USA), the suture instruments were passed through the lateral margin of the ruptured tendons, about 15 mm from the torn margin. In some cases, a suture hook was passed easily through a modified Neviaser portal and then we used this portal. Afterwards, at the time of lateral-row anchor insertion (5.0mm TwinFix anchors, Smith and Nephew Endoscopy, Andover, MA), the insertion point was selected by pulling the torn tendon margin to the greater tuberosity. If we could obtain a tight tension for the pulled tendon with a large cuff tear, we would insert the lateral anchor more medially, not just at the lateral margin of the greater tuberosity. Each suture was passed through the tendons at approximately 1 cm intervals. The number of anchors used was determined according to the size of the antero-posterior dimension of the torn cuff. In a moderate or large cuff tear, two – four anchors were inserted at 1 cm intervals. The sutures in the lateral row were sutured first, attached to the lateral margin of the greater tuberosity in a simple suture pattern so that the original footprint of the cuff could be restored. For cases where the antero-posterior length of the ruptured rotator cuff was narrow, we used the modified Mason-Allen technique as reported by Scheibel *et al*. 13 Where two sutures in the lateral row were tied. After restoration of the lateral surface of the footprint, by suturing the lateral row, the medial row sutures were then completed [Figure 3]. To obtain knot and loop security, horizontal mattress sutures were used in the medial row with the double lumen pusher (Surgeon’s Sixth finger; Arthrex, Naples, FL, USA). After completion of the medial and lateral row sutures, the stability of the repaired tendon was assessed by moving the shoulder through a complete range of motion.

After surgery, rehabilitation was carried out in the same manner for all patients; this included the arm in a sling for four weeks. Two days post surgery passive flexion of the shoulder joint and pendulum exercises were started. For four weeks after surgery, active assisted range of motion exercises using a bar was performed and an abduction brace applied. After four weeks, the pulley exercise was started and then strengthening exercises of the rotator cuff, scapular muscle stabilizing and posterior capsular stretching exercises were added gradually after six to eight weeks.

### Statistical analysis

Using the parametric t-test and nonparametric Wilcoxon rank sum statistics, we tested the statistical difference between the single row and the double row repair methods. The null hypothesis was that the distributions of the two methods were identical and the alternative hypothesis was that the two distributions differed only with respect to the median. We compared the clinical differences by age- and tear size- between the two groups. Statistical significance was considered when the *P* value was less than 0.05.

## RESULTS

The clinical outcomes of the arthroscopic single row repair group and the double row repair group were compared. The mean number of anchors used was 2.5 (range 1-5) and 3.2 (range 2-6) in group I and group II respectively. In Group I, the mean ASES score increased from 30.48 to 87.40 and the mean ASES score increased from 32.00 to 91.45 in the Group II. The mean UCLA score increased from the preoperative 12.23 to 30.82 in Group I and from 12.20 to 32.40 in Group II. For both methods, there was a significant improvement after repair compared to the preoperative state for the full thickness rotator cuff tears (*P*< 0.001). For both groups, no statistically significant differences with regard to forward flexion, abduction, external rotation and internal rotation were noted. The difference in the ASES score between the two groups was analyzed by the paired t-test and showed a *P* value of .247; the difference of the UCLA score analyzed by the paired t-test showed a *P* value of .237. Therefore, significant clinical diferences were not found in the comparisons between the two groups [Table 1]. We analyzed the data by age and cuff size tear. The size effect of the torn cuff was evaluated approximately by the parametric t-test and the nonparametric Wilcoxon signed rank test as well. However, no significant difference between repair methods for the tear size [Table 2] was noted. In addition, there were no significant differences between the two repair methods detected by patient age [Table 3]. Next, we evaluated differences between the groups using the performed t-test for each subtype of the UCLA scores. Here we found that the double row repair method showed better outcomes for strength and improved patient satisfaction [Table 4].

**Table 1:** Clinical difference between two repair methods (*P* value< 0)

Operation	Mean*	T	*P* value	WS**	Z	*P* value
ASES score	-3.33	-0.53	0.60	495.5	-0.6826	0.2474
UCLA score	-4.574	-0.96	0.34	494	-0.7157	0.2371

* Mean is the average of the differences between scores for post and pre operations

** WS is Wilcoxon rank sum statistic

**Table 2:** Summary statistics for difference between repair methods according to torn cuff size (*P* value<0.05)

Size	Mean*	T	*P* value
Large	-4.393	-0.38	0.7066
Medium	-1.882	-0.22	0.8316
Small	-12.6	-0.90	0.4034

* Mean is average of the differences between two operation effects

**Table 3:** Clinical difference between the operation methods for each age group with UCLA scores (*P* value< 0.05)

Age	Mean*	T	*P* value	WS**	Z	*P* value
less than 50	-1	-0.24	0.4102	14	-0.1302	0.4482
50~ 60	-0.636	-0.28	0.3896	124.5	-0.0988	0.4607
more than 60	-3.5	-1.42	0.0844	55.5	-1.2630	0.1033

* Mean is the average of the differences between scores for post and pre operations

** WS is Wilcoxon rank sum statistic

**Table 4:** Clinical difference between two methods according to the type in UCLA scores

Type	Mean*	T	*P* value	WS**	Z	*P* value
Pain	-1.237	0.22	0.4135	402	0.1373	0.4454
Function	-0.749	0.74	0.2329	563.5	0.7551	0.2251
Activity	-0.343	0.97	0.1688	569.5	0.9172	0.1795
Strength	0.0093	2.05	0.0232*	623.0	2.1386	0.0162*
Satisfaction	0.0017	1.95	0.0304*	590.0	1.7216	0.0426*

* Mean is the differences between the respective average UCLA scores of each operation method.

** WS denotes the Wilcoxon rank sum and *P* value of Wilcoxon is computed from the normal approximation for the one-sided alternative hypothesis

In group I, one patient suddenly developed shoulder pain after supervised physical therapy three weeks post-operatively. We evaluated the shoulder by MRI immediately and found that the rotator cuff was torn again. Revision surgery was performed immediately. In group II, two complications developed. One patient had continued mild shoulder pain for 11 months. At the second look arthroscopy, we found knot failure at the medial row of sutures due to the high tension at the repair site. However, the bone-tendon insertion surface was well healed at the lateral row suture area. In this case, we performed arthroscopic synovectomy and debridement and then the symptoms improved. The other complication occurred due to surgical technical errors. For the 1 cm sized small tear, the viewing field was so narrow at the time of medial anchor insertion, that the anchor was inserted incorrectly between the cuff tendon and subchondral bone of the humeral head. Using the double row repair method, revision surgery was performed after the removal of the anchor.

## DISCUSSION

The outcome of arthroscopic rotator cuff repair has gradually improved.^14^–^16^ However, despite the development of new repair techniques, the retear rate of repaired cuffs develops frequently.^17^–^19^ Torn cuff tissue is often degenerative and retracted; various factors are associated with the failure of rotator cuff repair.^20^,^21^ The arthroscopic single row repair method provides only the point of fixation compared to the double row repair. Recently, methods to reconstruct the rotator cuff footprint, by the double-row repair method, have been proposed.^3^ Currently, the double row repair method has been widely adopted for cuff repair.^8^,^9^,^22^

Initially, Alpreleva *et al*.^4^ introduced the traditional trans-osseous repair method or the arthroscopic single row repair method, which does not reconstruct the original footprint. Meier *et al*.^7^ reported that the double-row repair method might be superior to other techniques for initial repair strength. Park *et al*.^23^ reported that in comparison with the suture anchor method, the trans-osseous repair method increases the contact surface more, and thus the distribution of pressure is better, resulting in stronger and faster healing in the footprint area. Milano *et al*.^24^ reported that the double row technique recovered with a larger footprint and that the biological quality of the tendon repair could be improved. Kim *et al*.^5^ reported that the footprint reconstruction of the rotator cuff, using a double-row repair, improved the initial strength and stiffness; in addition, it decreased gap formation and strain over the footprint when compared with the single-row repair. Other mechanical studies showed that the double-row technique produced the greatest contact area and the second-highest contact pressure.^25^

Contrary to the biomechanical studies, there are debates about clinical outcomes when the single and double row repair techniques are compared.^22^ Fealy *et al*.^26^ reported that in 75 patients, using the mini-open double row fixation method, small, moderate and large tears were sutured and good results were reported. Sugaya *et al*.^22^ retrospectively studied 80 shoulders in consecutive patients who had undergone rotator cuff repairs (41 double row and 38 single row). At a mean follow- up of 35 months (range, 24 to 60 months), there was no difference between the groups based on the ASES and UCLA rating scales. MRI evaluation of cuff integrity did show better structural outcomes with the double row repair in small- to medium-sized tears (*P*< .05). In addition, they found a significant difference in the postoperative strength scores of the UCLA score between the two groups. Anderson *et al*.^8^ reported that the arthroscopic two-row rotator cuff repair produces excellent functional outcomes and repair integrity comparable with previously reported open repairs.

Similarly, in our study the two measurement scores, the ASES and UCLA scores, result in similar clinical outcomes for the two techniques. According to the age and size of the torn cuff, there was no significant difference between the two methods. However, two subtest UCLA scores were significantly different when the two groups were compared. That is, the double row repair method resulted in greater strength and improved patient satisfaction. Strength is known to influence patient satisfaction after rotator cuff repair.^21^,^27^ Although this study showed no preference for either one of the two repair methods, greater satisfaction of the patients and restoration of muscle power favored the double row repair method. For optimal results, tendon repairs require initial tension-free or low-tension fixation. If this is not possible and high tensions are imposed, the repair will be overloaded and the fixation will fail.^28^ One patient in the double row technique group, had knot failure in the medial row of sutures due to the high tension at the repair site; but the bone-tendon insertion surface was well healed at the lateral row suture area. This situation can be prevented if the initial repair is tension free.

The double row repair technique requires a longer surgical time and is a technically more demanding procedure. However, the time difference between the two procedures is about 30 minutes in our study; further, we believe that the surgeon can reduce surgical time by improving his or her technical skills, which is primarily a learning curve issue. Another concern regarding arthroscopic double row repair is its cost. We believe that the benefit of arthroscopic repair for patients more than overcomes the cost issue if we can provide better structural and functional outcomes with higher patient satisfaction. The most important limitation of our study was the retrospective study design and the fact that we evaluated the clinical results at the last follow up visit. In addition, there were a small number of patients in each subgroup (small, medium, large tear) because we excluded partial, massive and irreparable cuff tears and subscapularis and biceps tendon tears. Different from other studies, we did not obtain postoperative imaging. Strength evaluation can be considered an indirect measurement of tendon healing, because it has been significantly correlated with structural integrity of the rotator cuff.^16^ Patients were followed for only a period of two years. This timeframe showed the stabilization of symptoms but longer follow up is needed for both groups for more definitive results. In summary the two repair methods, single row and double row repair techniques, showed good clinical results for full thickness rotator cuff tears. However, when comparing the two methods, the double row repair group showed better clinical results for strength recovery and patient satisfaction.
